# Spring cold stress at high altitudes in southeastern Xizang activates *CsABF2* to regulate chlorophyll degradation and phenolic biosynthesis in tea plants

**DOI:** 10.1093/hr/uhaf279

**Published:** 2025-10-22

**Authors:** Yipeng Huang, Didi Jin, Tianming Jiao, Zhenhong Wang, Ting Jiang, Lei Zhao, Xiaolan Jiang, Haiyan Wang, Yajun Liu, Yunsheng Wang, Liping Gao, Tao Xia

**Affiliations:** State Key Laboratory of Tea Plant Germplasm Innovation and Resource Utilization/Key Laboratory of Tea Biology and Tea Processing of Ministry of Agriculture/Anhui Provincial Laboratory of Tea Plant Biology and Utilization, Anhui Agricultural University, West 130 Changjiang Road, Hefei 230036, Anhui, China; State Key Laboratory of Tea Plant Germplasm Innovation and Resource Utilization/Key Laboratory of Tea Biology and Tea Processing of Ministry of Agriculture/Anhui Provincial Laboratory of Tea Plant Biology and Utilization, Anhui Agricultural University, West 130 Changjiang Road, Hefei 230036, Anhui, China; State Key Laboratory of Tea Plant Germplasm Innovation and Resource Utilization/Key Laboratory of Tea Biology and Tea Processing of Ministry of Agriculture/Anhui Provincial Laboratory of Tea Plant Biology and Utilization, Anhui Agricultural University, West 130 Changjiang Road, Hefei 230036, Anhui, China; College of Resources and Environment, Xizang Agricultural and Animal Husbandry University, 100 Yucai West Road, Linzhi 860000, Xizang, China; State Key Laboratory of Tea Plant Germplasm Innovation and Resource Utilization/Key Laboratory of Tea Biology and Tea Processing of Ministry of Agriculture/Anhui Provincial Laboratory of Tea Plant Biology and Utilization, Anhui Agricultural University, West 130 Changjiang Road, Hefei 230036, Anhui, China; College of Horticulture, Qingdao Agricultural University, 700 Changcheng Road, Qingdao 266109, Shandong, China; State Key Laboratory of Tea Plant Germplasm Innovation and Resource Utilization/Key Laboratory of Tea Biology and Tea Processing of Ministry of Agriculture/Anhui Provincial Laboratory of Tea Plant Biology and Utilization, Anhui Agricultural University, West 130 Changjiang Road, Hefei 230036, Anhui, China; School of Life Science, Anhui Agricultural University, 130 Changjiang West Road, Hefei 230036, Anhui, China; School of Life Science, Anhui Agricultural University, 130 Changjiang West Road, Hefei 230036, Anhui, China; School of Life Science, Anhui Agricultural University, 130 Changjiang West Road, Hefei 230036, Anhui, China; School of Life Science, Anhui Agricultural University, 130 Changjiang West Road, Hefei 230036, Anhui, China; State Key Laboratory of Tea Plant Germplasm Innovation and Resource Utilization/Key Laboratory of Tea Biology and Tea Processing of Ministry of Agriculture/Anhui Provincial Laboratory of Tea Plant Biology and Utilization, Anhui Agricultural University, West 130 Changjiang Road, Hefei 230036, Anhui, China

## Abstract

The tea plant (*Camellia sinensis*), native to warm and humid low-latitude regions of southwestern China, has expanded to higher altitudes, including southeastern Xizang, where cultivation above 2500 m poses challenges due to low accumulated temperatures. However, the impact of high-altitude climatic conditions, particularly temperature, on tea growth remains underexplored. To investigate, weather stations were deployed at three altitudes in southeastern Xizang to monitor spring temperature fluctuations: Medog (MD, 1200 m), Zayü (ZY, 1720 m), and Layue in Bayi District (BY, 2600 m). Field observations and meteorological data indicated that the milder spring temperatures in MD and ZY facilitated normal budburst and growth, whereas the lower temperatures in BY delayed budburst and resulted in leaf yellowing and browning. Comparative experiments revealed that seedlings exposed to fluctuating low temperatures (10°C/4°C) experienced the most severe cold injury and exhibited the lowest germination rates compared to seedlings under constant-temperature treatments. Transcriptome analysis uncovered differential expression of genes involved in chlorophyll degradation, lignin biosynthesis, and flavonoid pathways under cold stress. Functional characterization of the cold-induced transcription factor *CsABF2* revealed its central role in activating these pathways, as evidenced by antisense oligodeoxynucleotide (AsODN) silencing and promoter activation assays, to activate key downstream genes: *CsSGR1* (chlorophyll degradation), *CsPALa* (phenylpropanoid pathway), and *CsMYB6c* (flavonoid biosynthesis). These results provide mechanistic insights into how spring temperature variability at high altitudes impairs tea plant development and alters quality-related metabolites, offering a molecular basis for improving cold resilience in tea cultivation.

## Introduction

The tea plants, originally adapted to southwestern China, thrives in warm, humid, and shaded environment [1, 2]. Through cultivation expansion, tea has gradually migrated to higher latitudes and altitudes, undergoing adaptive changes in response to progressively colder climates [3]. Climatic factors, particularly temperature, play multifaceted roles in influencing the survival of tea plants and the accumulation of quality-related components [4–7]. From the perspective of germplasm, the primary concern is the impact of low temperatures in winter on plant survival. Tea cultivars vary significantly in cold tolerance and can endure temperatures as low as −10 to −15°C [8]. Also, cultivation has even extended as far north as 49°N in Ukraine [9]. Another important consideration is the effect of low temperatures in spring on bud burst timing and the development of quality traits.

Early spring temperatures around 0°C can lead to cold damage or frost injury and pose serious challenges to tea growth, especially during cold snaps following premature warming [6, 10, 11]. While extensive research has been conducted on the physiological and molecular mechanisms of cold stress and low-temperature adaptation in tea plants [7, 12–14], factors such as the duration of low temperatures (above 0°C) in early spring, large diurnal temperature variations, and slow warming rates in high-latitude and high-altitude regions have received little attention, despite their significant impact on tea production.

Southeastern Xizang, influenced by warm, humid air currents from the Indian Ocean and characterized by its complex terrain of mountainous river valleys, exhibits a significant altitudinal gradient [15]. Variations in altitude bring about significant changes in multiple environmental factors, including temperature, humidity, oxygen availability, and light intensity [16, 17]. These environmental gradients impose considerable challenges on plant growth and survival. In high-altitude environments, plants have developed various mechanisms to cope with the adverse conditions present in such settings. Morphologically, most alpine plants exhibit thickened waxy cuticles on their leaves to mitigate damage caused by low temperatures, suppression of vegetative growth, increase in stomatal conductance, enhancement of photosynthetic efficiency, and dwarfing to minimize exposure to wind and cold [16]. Physiologically, alpine plants enhance the production of osmoregulatory substances to prevent damage from freezing or ice crystal formation [18]. High-altitude plants possess a stronger reactive oxygen species scavenging capacity, and the elevation-dependent increase in proline and soluble sugar contents suggests that these compounds play an important role in osmotic regulation [19].

To adapt to environmental stresses, plants enhance their secondary metabolism as a defense strategy [16]. Phenolic compounds, such as flavonoids and phenolic acids, increases with elevation [20, 21]. The mechanisms through which certain novel flavonoid biosynthesis gene clusters modulate flavonoid production and enhance adaptation to high-altitude environments [22]. Additionally, some alpine plants enhance the accumulation of B-ring trihydroxy anthocyanins, deepening leaf or flower coloration to prevent ultraviolet radiation damage [23]. Moreover, terpenoids, including monoterpenes, sesquiterpenes, diterpenes, and triterpenes and as well as volatile secondary metabolites, accumulate significantly under high-altitude conditions [24–26]. In summary, these integrated physiological and morphological strategies reflect long-term evolutionary adaptations to high-altitude environments and provide significant insights into the potential mechanisms of ecological resilience in plants. In summary, these integrated morphological strategies reflect long-term evolutionary adaptations to high-altitude environments.

Tea plants cultivated at higher altitudes in southeastern Xizang display adaptive traits, including a shortened growth period and reduced survival rates of cuttings in early spring. The tea plants cultivated in this region are subjected to diverse ecological conditions, providing valuable material for studying the effects of environmental differences on tea plant growth, development, and the accumulation of quality-related components. Furthermore, this serves as a basis for investigating how high-altitude environments affect the aroma and taste characteristics of tea plant [27].

This study, based on monitoring data provided by the National Tibetan Plateau/Third Pole Environment Data Center, as well as observations from miniature weather stations and records from tea cultivation, examines how temperature variations in spring across different altitudes in southeastern Xizang affect the growth of tea seedlings. Additionally, by simulating low-temperature conditions during early spring in a controlled climate chamber, we employed transcriptomic and molecular biology techniques to investigate the causes of cold-induced leaf damage and the functional role of the cold-responsive gene *CsABF2*. In summary, the *CsABF2* transcription factor induced by low temperature not only promotes the degradation of chlorophyll in tea leaves but also facilitates the biosynthesis of lignin and anthocyanins. This study enhances our understanding of the effects of low temperatures at high altitudes on the yellowing of tea leaves and the accumulation of secondary metabolites.

## Results 

### Climatic characteristics of southeastern Xizang and cold injury in spring for tea plants

**Figure 1:**
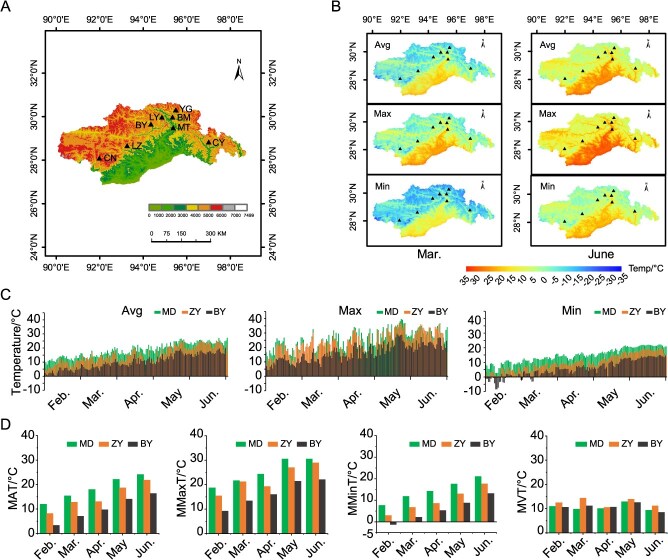
Climatic conditions suitable for tea growth in southeastern Xizang. (A) DEM of tea cultivation regions in southeastern Xizang. The map was obtained from the Tianditu website (https://www.tianditu.gov.cn, Map Approval Number: GS (2024)0650). Tea cultivating locations in southeastern Xizang include Medog county (MD), Bomi county (BM), Zayü county (ZY), Bayi district (BY), Cuona county (CN), Longzi county (LZ), Layue (LY), and Yigong tea garden (YG); (B) spatial distribution of temperature in March and June (2014–2023) across tea-cultivating areas in southeastern Xizang, temperature includes average temperature (Avg), maximum temperature (Max), and minimum temperature (Max); (C) diurnal variation of average air temperature in spring 2024 at the three tea gardens in southeastern Xizang; and (D) monthly variation of average air temperature in spring 2024 at the three tea gardens, monthly average temperature (MAT), monthly maximum temperature (MMaxT), monthly minimum temperature (MMinT), and monthly variation temperature (MVT).

This study first downloads the GeoJSON format data corresponding to the required administrative divisions from the official Tianditu website (https://www.tianditu.gov.cn, Map Approval Number: GS (2024)0650), and the map with the approval number is shown in Fig. S1. Subsequently, the JSON format data is converted into ESRI Shapefile (.shp) using Mapshaper (https://mapshaper.org). After completion of the conversion, the shapefile is imported into ArcMap 10.8.1 for further processing. Finally, digital elevation models (DEMs) are constructed for Nyingchi city and Shannan city in Xizang, enabling effective visualization of the locations of tea plantations situated in southeastern Xizang (Fig. 1A). The map reveals that tea cultivation in southern Xizang is primarily distributed between 28°N–31°N latitude and 92°E–97°E longitude. Among these regions, the tea gardens in Medog (MD) are mostly located at the lowest altitudes (average altitude of ~1200 m) and are strongly influenced by warm, humid air masses from the Indian Ocean [28, 29]. Consequently, this area features a low-mountain subtropical humid climate. In contrast, the tea-cultivating region of Zayü (ZY) lies at the confluence of the Himalayas and the Hengduan Mountains, with altitudes ~1700 m. This area experiences a subtropical mountain humid monsoon climate characterized by mild winters, hot summers, and consistently humid, rainy conditions throughout the year. The other tea-cultivating regions are situated at average altitudes above 2000 m and are subtropical mountainous areas characterized by a semi-humid, warm temperate monsoon climate. Among them, Layue in Bayi District (BY) is located at the highest altitude, exceeding 2600 m.

Monthly average temperature, minimum temperature, and maximum temperature datasets with a spatial resolution of 1 km were obtained from the National Tibetan Plateau/Third Pole Environment Data Center (https://data.tpdc.ac.cn) [30–33]. With ArcMap 10.8.1, spatial distribution maps of average, minimum, and maximum temperatures for March and June over the past decade (2014–2023) in southeastern Xizang were generated (Fig. 1B). The results indicate that the low-altitude regions of MD and ZY exhibit higher monthly average, minimum, and maximum temperatures compared to the high-altitude regions in both March and June. Additionally, the data reveal that some valley areas within the high-altitude regions experience higher temperatures than the surrounding mountainous terrain.

In most tea-cultivating regions of the Northern Hemisphere, tea buds typically begin to sprout between March and April each year. An average spring temperature of 10°C is generally considered the threshold for budburst. During this critical period, low monthly minimum temperature (MMinT) or sudden extreme cold events can lead to chilling or freezing injury. In order to closely examine variations in springtime temperatures during the budding season across tea gardens at different altitudes, compact weather stations were installed in tea gardens at MD, ZY, and BY tea. These stations recorded daily fluctuations in air temperature from February to June 2024 (Fig. 1C; Table S1), along with variations in monthly average temperature (Fig. 1D). The results showed a clear trend of decreasing monthly mean temperature (MAT), monthly maximum temperature (MMaxT), and MMinT with increasing altitude. In March, the low-altitude MD garden recorded a monthly minimum average temperature of 11.89°C, a maximum of 21.72°C, and an average temperature of 15.36°C. The ZY garden recorded a minimum of 6.80°C, a maximum of 21.24°C, and an average of 12.78°C. In contrast, the BY Garden at high altitude had MMinT only 2.23°C, MMaxT was 13.44°C, and MAT was 7.12°C. These findings indicate that budburst occurs earliest in the MD garden and latest in the BY garden. Based on temperature patterns, it can be inferred that budburst in the high-altitude garden at BY likely occurs after May and is highly susceptible to freezing injury.

In order to observe the effect of high-altitude climate on the growth and development of tea plants, in 2022, we planted 20 one-year-old tea plant varieties from Anhui, Zhejiang, and Fujian at three distinct altitudes in Xizang: low-altitude region ZY (1720 m), and high-altitude Yigong (YG, 2200 m), and BY (2600 m). Through comparative cultivation experiments, this study aimed to investigate whether the high-altitude climate induces frost damage in tea plants and to identify tea plant varieties that are suitable for cultivation in these conditions. The growth of the seedlings was assessed in mid-May two years later, and images of seven cultivars were selected for presentation (Fig. S2A). Compared with the vigorous growth observed in ZY, seedlings at the higher altitude sites (YG and BY) exhibited symptoms across all cultivars. Specifically, the young apical leaves showed uneven yellowing, while the older basal leaves developed browning. These symptoms gradually subsided as temperatures increased (Fig. S2B). In summary, these observations suggest that the slow rise in spring temperatures (April–May) at high altitudes is likely a major environmental factor limiting the growth of tea plants in this region.

According to the variation in diurnal temperature (Fig. 1C), the minimum temperature in the high-altitude region of BY often falls below 10°C, and the average difference between daily maximum and minimum temperatures reaches ~10°C. Based on this, we hypothesize that large fluctuations in diurnal temperatures may be a primary cause of cold injury in tea plants grown at high altitudes. To test whether low temperatures (4–10°C) contribute to cold injury in tea plants, we designed three controlled day/night temperature treatments: 4°C/4°C (constant low temperature), 10°C/4°C (diurnal fluctuation), and 10°C/10°C (constant moderate temperature). As shown in Fig. S2C, no symptoms of cold injury were observed in the 10°C/10°C group. In contrast, both the 4°C/4°C and 10°C/4°C treatments resulted in water-soaked lesions characteristic of cold injury. In addition, leaf browning was more significant in the 10°C/4°C treatment than in the 4°C/4°C treatment.

All three groups of treated tea seedlings were maintained at room temperature to observe bud development and assess the impact of cold injury on the yield. As shown in Figs S2D and E, compared with seedlings subjected to the 10°C/10°C treatment, those exposed to 4°C/4°C and 10°C/4°C exhibited significantly reduced weights of fresh shoots (one bud with two leaves) at both the first (15 days at room temperature) and the second (30 days at room temperature) harvests. Notably, the damage from the 10°C/4°C treatment persisted into the second sampling. These findings suggest that although the temperature difference (6°C) was relatively small, diurnal fluctuations between 4 and 10°C caused more severe damage to tea plants than other treatments. This further supports the idea that in high-altitude regions such as BY, day–night temperature fluctuation during the spring (April–May) has a significant impact on the growth of tea plants.

### Molecular basis of spring regional yellowing leaf in high-altitude tea plants

**Figure 2:**
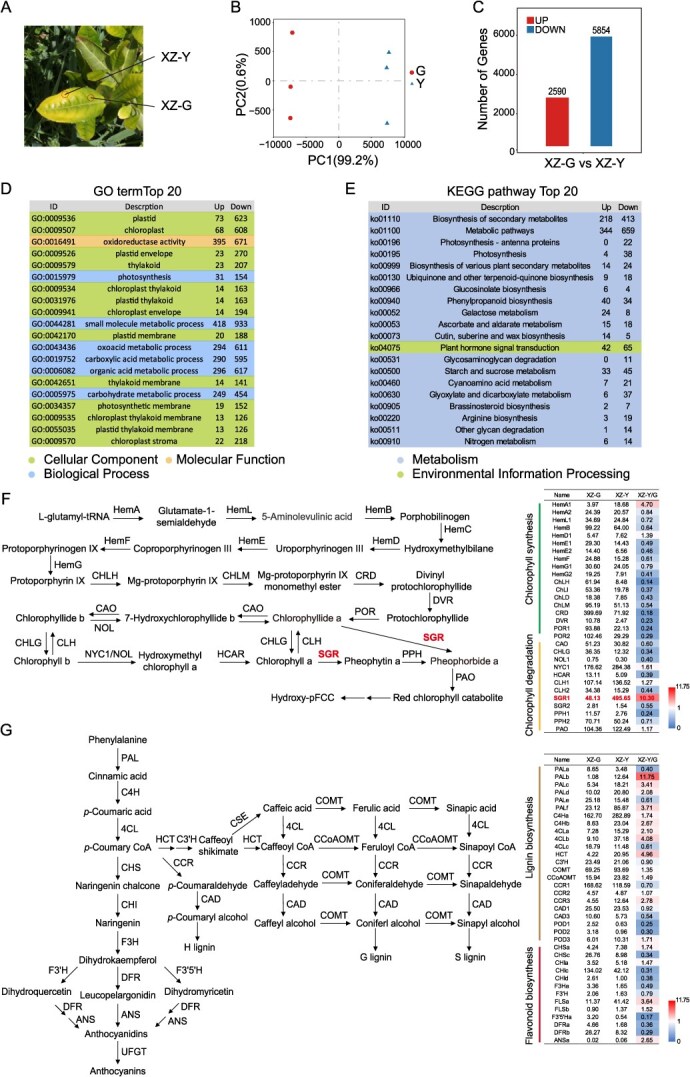
Transcriptomic insights into leaf yellowing in tea plants grown at high altitudes in Xizang: (A) XZ-Y and XZ-G indicate different leaf areas, respectively; (B) principal component analysis (PCA) of transcriptome profiles; (C) number of differentially expressed genes (DEGs) identified between yellow and green leaf regions; (D) Gene Ontology (GO) enrichment analysis of DEGs; (E) Kyoto Encyclopedia of Genes and Genomes (KEGG) pathway enrichment analysis of DEGs; (F) DEGs in pathways of chlorophyll biosynthesis and degradation; and (G) DEGs in pathways of phenylpropanoid and lignin biosynthesis.

In early spring, tea plants in the high-altitude regions of southeastern Xizang exhibit reddening of mature leaves and uneven region-specific yellowing young leaves. To explore the molecular mechanism underlying the regional yellowing tender shoots, this study employed transcriptome sequencing to compare the gene expression profiles between XZ-Y and XZ-G areas of the third leaf from the apical shoot (Fig. 2A).

Principal component analysis (PCA) of the transcriptome data showed clear clustering within groups and distinct separation between groups, indicating good intra-group reproducibility and significant inter-group differences (Fig. 2B). Based on screening criteria of FDR < 0.05 and |log₂ fold change| > 1, a total of 2590 genes were identified as upregulated and 5854 as downregulated in the green area relative to the yellow area (Fig. 2C). GO enrichment analysis revealed that the differentially expressed genes (DEGs) were primarily enriched in categories such as plastid (GO:0009536; GO:0009507), plastid envelope (GO:0009526), small molecule metabolic processes (GO:0044281), oxoacid metabolism (GO:0043436), and organic acid metabolism (GO:0006082) (Fig. 2D; Table S2). KEGG pathway analysis showed significant enrichment in secondary metabolism (ko01110) and general metabolic pathways (ko01100), particularly those related to photosynthesis (ko00195), ubiquinone and terpenoid–quinone biosynthesis (ko00130), and phenylalanine metabolism (ko00940) (Fig. 2E; Table S3).

Further analysis of gene expression in the pathways related to chlorophyll metabolism revealed that several genes involved in chlorophyll degradation, Non-Yellow Coloring 1 (*NYC1*), Chlorophyllase (*CLH*), Pheophorbide a oxygenase (*PAO*), and Stay-Green 1 (*SGR1*), were significantly upregulated in the yellow-spotted areas compared to the green regions, with *CsSGR1* exhibiting a 10.3-fold increase (Fig. 2F). According to previous studies, *CsSGR1* is crucial in chlorophyll degradation, as plants lacking *SGR1* exhibit a stay-green phenotype [34]. Additionally, the study examined gene expression in the phenylpropanoid and lignin biosynthesis pathways (ko00940). The expression of phenylalanine ammonia lyase (*PAL*), cinnamate 4-hydroxylase (*C4H*), 4-coumaroyl-CoA ligase (*4CL*), and shikimate/quinate hydroxy cinnamoyl transferase (*HCT*) was significantly higher in the region-specific yellowing leaves, especially in the upstream segment of the phenylalanine metabolic pathway (Fig. 2G).

### Transcriptomic and metabolic responses of tea plants to indoor low-temperature treatment

**Figure 3:**
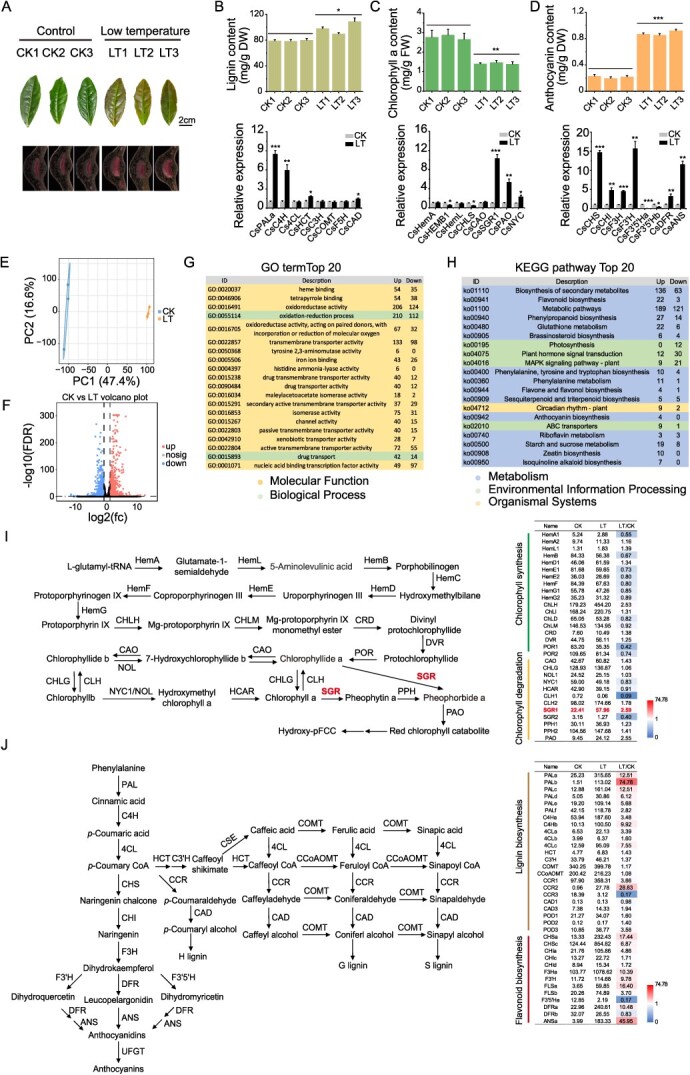
Effects of indoor low-temperature treatment on gene expression and metabolism in tea seedlings. (A) Phenotypic variation in the third leaf of tea seedlings after three weeks of low-temperature treatment, including differences in lignin content. Lignin content was visualized by phloroglucinol staining of leaf cross-sections; (B) quantification of lignin content and expression levels of lignin biosynthesis-related genes; (C) chlorophyll content and expression levels of chlorophyll-related genes; (D) anthocyanin content and expression levels of anthocyanin biosynthesis genes; (E) PCA of transcriptome profiles under different temperature conditions; (F) volcano plotting of DEGs between control groups and low-temperature; (G) GO enrichment analysis of DEGs; (H) KEGG enrichment analysis of DEGs; (I) differential expression of genes in pathways of chlorophyll biosynthesis and degradation; and (J) differential expression of genes in pathways of phenylpropanoid and lignin biosynthesis. All data are presented as the mean ± standard deviation (SD) of three biological replicates (*n* = 3). Statistical significance was determined by the Student’s *t*-test, with ^*^P < 0.05, ^**^P < 0.01, and ^***^P < 0.001.

To investigate the molecular basis of cold-induced color changes in tea seedlings under low diurnal temperatures, this study employed transcriptome sequencing to analyze the impact of normal diurnal temperatures (25°C/20°C) versus low-temperature stress (10°C/4°C) on gene expression profiles in tea seedlings (Fig. 3A). Quantitative analysis of lignin, chlorophyll, and anthocyanins in seedling leaves revealed that low-temperature treatment significantly altered the metabolic characteristics of tea plants. With phloroglucinol-HCl staining to detect lignin distribution, the low-temperature group exhibited a more intense red coloration, indicating a significantly higher lignin accumulation compared to the control group (Fig. 3A). Further quantitative analysis confirmed that low-temperature treatment not only increased the total content of lignin but also significantly upregulated the expression of key genes in the phenylpropanoid-lignin pathway, i.e., *CsPALa* and *CsC4H* (Fig. 3B). In terms of pigment metabolism, low-temperature treatment led to a significant reduction in the content of chlorophyll a in tea leaves, accompanied by significant upregulation of *CsSGR1* and *CsPAO*, genes involved in chlorophyll degradation (Fig. 3C). Also, genes associated with anthocyanin biosynthesis, chalcone synthase (*CHS*), chalcone isomerase (*CHI*), flavanone 3-hydroxylase (*F3H*), dihydroflavonol 4-reductase (*DFR*), and anthocyanidin synthase (*ANS*), were all significantly upregulated in the low-temperature group, resulting in a significant increase in anthocyanin content compared to the control group (Fig. 3D).

Transcriptome sequencing was performed on two groups of samples, and PCA was used to assess the distribution patterns of the data. The analysis revealed that samples within each group were tightly clustered, while those between groups were clearly separated, indicating high intra-group consistency and significant differences between treatments (Fig. 3E). Based on the thresholds of FDR < 0.05 and |log₂FC| > 1, the volcano plot analysis identified 1220 genes as significantly upregulated and 1275 genes as downregulated under low-temperature treatment (Fig. 3F). GO enrichment analysis indicated that among the top 20 enriched terms, 18 were classified under ‘molecular function’ and 2 under ‘biological process.’ Notably, ‘oxidoreductase activity’ (GO:0016491), and ‘oxidation–reduction process’ (GO:0055114) were significantly enriched (Fig. 3G; Table S4). KEGG pathway analysis further revealed that DEGs were predominantly enriched in pathways related to secondary metabolism (ko01110) and primary metabolism (ko01100), specifically involving flavonoid biosynthesis (ko00941), glutathione metabolism (ko00480), phenylalanine metabolism (ko00940), photosynthesis (ko00195), and plant hormone signal transduction (ko04075) (Fig. 3H and Table S5). These results suggest that low-temperature stress may influence photosynthetic efficiency in tea plants and modulate the biosynthetic pathways of lignin and flavonoids.

This study further explored the expression characteristics of genes involved in chlorophyll biosynthesis, phenylpropanoid metabolism, and lignin biosynthesis. Among the genes associated with chlorophyll degradation, *CsNYC1*, *CsCLH1*, *CsPAO*, and *CsSGR1* exhibited significantly elevated transcript levels in the yellow-spotted region compared to the green region (control), with *CsSGR1* exhibiting the most significant upregulation, 2.59 times higher than the control (Fig. 3I). Similarly, key genes involved in the pathways of phenylpropanoid and lignin biosynthesis, *CsPALb*, *CsC4Hb*, *Cs4CLc*, and *CsCCR2*, were significantly upregulated, particularly in the early stages of the phenylalanine metabolism, with *CsPALb* exhibiting the most substantial increase in expression (Fig. 3J). Notably, the change trend of expression levels of some genes related to chlorophyll synthesis and degradation, and lignin synthesis was consistent between samples from cold-stressed tea plants in Xizang plantations and laboratory-grown seedlings subjected to low-temperature treatment.

### Functional characterization of the cold-induced gene *CsABF2*

#### Identification and expression analysis of *CsABF2* as a potential regulator of *CsSGR1* in tea plants

**Figure 4:**
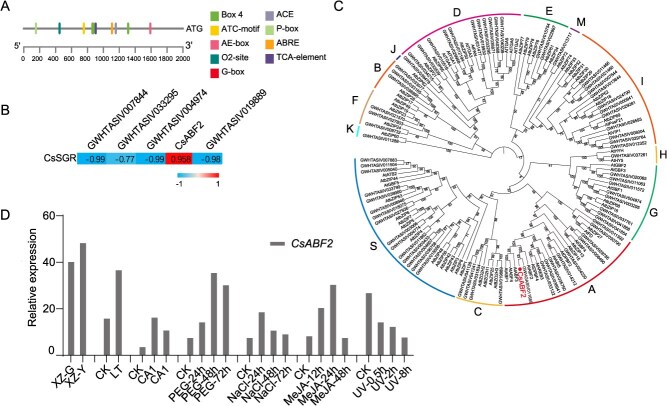
Identification and expression profiling of *CsABF2* in tea plants. (A) Cis-regulatory element analysis of the *CsSGR1* promoter; (B) correlation analysis between the expression of *bZIP* transcription factors and *CsSGR1*; (C) phylogenetic analysis of *CsABF2* and its homologs in other plant species; (D) expression of *CsABF2* in response to low temperature, PEG-induced drought, salt stress, MeJA treatment, and UV exposure based on publicly available transcriptome datasets (Tea Plant Information Archive (TPIA)).

As demonstrated above, the region-specific yellowing observed in tea leaves during spring in high-altitude regions of Xizang is primarily caused by low-temperature conditions. To investigate the molecular basis of this phenomenon, this study focused on identifying transcription factors that regulate chlorophyll content in tea leaves. An analysis of the functional regulatory elements in the promoter region of *CsSGR1* identified several key elements associated with light signaling and plant hormone responses (Fig. 4A). Specifically, Box 4, AE-box, and ATC-motif are involved in light responsiveness; G-box and ACE also function in light signaling; ABRE is associated with abscisic acid (ABA) signaling; TCA-element is linked to salicylic acid signaling; and P-box is related to gibberellin response. Previous research has shown that ABF transcription factors, which belong to subgroup A of the bZIP family, are crucial in regulating ABA-mediated abiotic stress responses in plants [35, 36]. In the model plant *Arabidopsis thaliana*, members such as *ABF2, ABF3*, and *ABF4* significantly regulate ABA-induced chlorophyll degradation and leaf senescence [34]. Based on expression correlation analysis, the expression of bZIP transcription factors exhibited a strong positive correlation with *CsSGR1* expression (Fig. 4B; Table S6). Phylogenetic analysis using MEGA7 revealed that the identified *CsbZIP* is homologous to *AtABF2*, an ABA-responsive element-binding factor in Arabidopsis, and belongs to the ABF subgroup of the bZIP family (Fig. 4C). Therefore, it was designated as *CsABF2*. Analysis of publicly available transcriptome datasets from the TPIA and NCBI database (Fig. 4D) showed that *CsABF2* expression was significantly altered under various stress conditions. Cold treatment, PEG-induced drought, salt stress, and MeJA application all significantly upregulated *CsABF2* expression, whereas UV radiation suppressed it. These results demonstrate that *CsABF2* is a stress-responsive gene.

#### *CsABF2* expression is upregulated by low-temperature and ABA treatments

**Figure 5:**
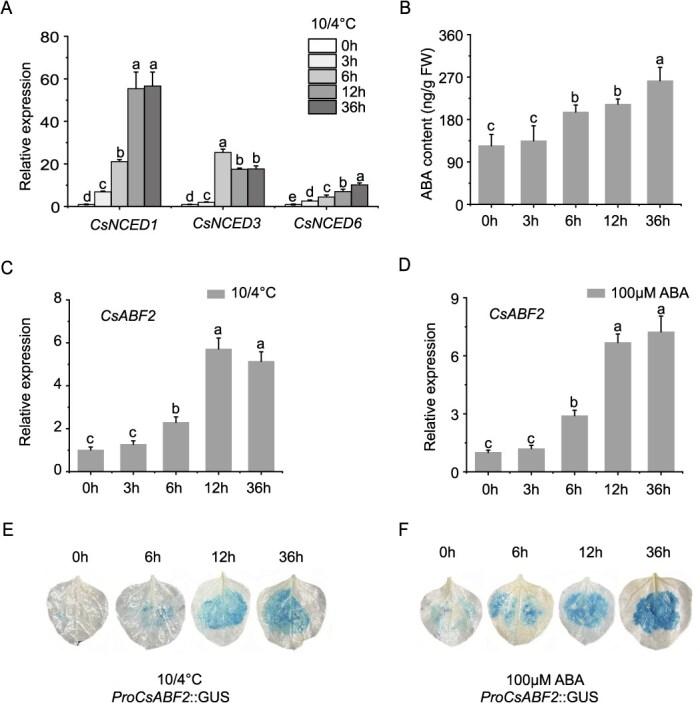
Induction of *CsABF2* expression and promoter activity by low temperature and ABA. (A) Low-temperature treatment upregulates the expression of *CsNCED1*, *CsNCED3*, and *CsNCED6*, key genes involved in ABA biosynthesis; (B) low-temperature treatment promotes ABA accumulation; (C) low-temperature treatment enhances *CsABF2* transcript levels; (D) exogenous application of 100 μM ABA promotes *CsABF2* expression; (E) low temperature activates *CsABF2* promoter activity, as shown by GUS staining in *N. benthamiana*; (F) ABA treatment also induces *CsABF2* promoter activity in *N. benthamiana.* All data are presented as the mean ± standard deviation (SD) of three biological replicates (*n* = 3). Different letters indicate statistically significant differences at *P* < 0.05, based on Tukey’s honestly significant difference (HSD) test.

As a key regulatory enzyme in ABA biosynthesis, 9-*cis*-Epoxycarotenoid Dioxygenase (*NCED*) is crucial [37, 38]. In plants, ABA levels are induced in response to various stress signals, primarily through the upregulation of genes encoding enzymes involved in ABA biosynthesis [39]. Among these, *NCED* is considered to catalyze the rate-limiting step in ABA production [40]. Previous studies have reported that the expression of *NCED* and *ABA2* is upregulated when tea plants are exposed to cold stress [39]. To investigate the involvement of *CsABF2* in ABA signaling under cold stress, we analyzed the expression of *CsNCEDs* gene and ABA accumulation. The results indicated that cold treatment led to an upregulation of *CsNCED1*, *CsNCED3*, and *CsNCED6*, all of which are closely associated with ABA biosynthesis, accompanied by a continuous increase in ABA levels (Fig. 5A and B). Further molecular analysis revealed that both cold stress and exogenous ABA treatment significantly increased the transcriptional level of *CsABF2* (Fig. 5C and D). Promoter activity assays in *Nicotiana benthamiana* further confirmed this response, where GUS staining showed that *CsABF2* promoter activity increased in a time-dependent manner following cold and ABA treatments (Fig. 5E and F). The mechanism of *CsABF2* gene expression stimulated by low temperature and ABA requires further investigation.

#### Functional validation of *CsABF2* target genes via antisense oligodeoxynucleotide-mediated gene silencing

**Figure 6:**
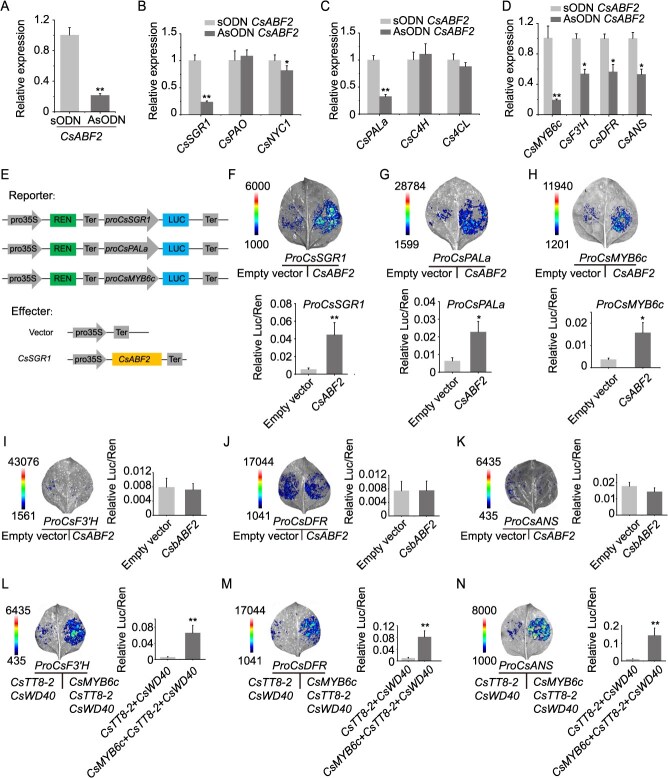
*CsABF2* activates genes involved in chlorophyll degradation, the phenylpropanoid pathway, and anthocyanin biosynthesis. (A–D) AsODN-mediated knockdown of *CsABF2* in tea leaves: (A) relative expression level of *CsABF2* following AsODN treatment; (B) transcript levels of chlorophyll degradation–related genes (*CsSGR1*, *CsNYC1*, and *CsPAO*) after *CsABF2* silencing; (C) expression changes in phenylpropanoid pathway–related gene *CsPALa*, *CsC4H*, and *Cs4CL*; (D) expression of flavonoid/anthocyanin biosynthetic genes (*CsMYB6c*, *CsF3′H*, *CsDFR*, and *CsANS*) after *CsABF2* suppression. (E–H) Dual-luciferase reporter assays for transcriptional activation by *CsABF2*: (E) schematic diagram of effector and reporter construct design; (F–H) activation of *CsSGR1*, *CsPALc*, and *CsMYB6c* promoters by *CsABF2*, shown by increased Luc/Ren ratios in *N. benthamiana*. (I–N) Regulatory cascade involving *CsABF2*, *CsMYB6c*, and *CsTT8–2*; (I–K) dual-luciferase assays showing activation of *CsMYB6c* and *CsTT8–2* by *CsABF2*; (L–N) promoter activation of *CsF3′H*, *CsDFR*, and *CsANS* by *CsMYB6c*, *CsTT8-2,* and *CsWD40*, as indicated by Luc/Ren activity. All data are presented as the mean ± SD of three biological replicates (*n* = 3). Statistical significance was determined by the Student’s *t*-test, with ^*^P < 0.05 and ^**^P < 0.01.

Antisense oligodeoxynucleotide (AsODN) technology is an effective tool for suppressing gene expression and investigating gene function [41, 42]. In this study, AsODN-mediated knockdown of *CsABF2* was conducted in tea leaves, resulting in a significant reduction in *CsABF2* expression (Fig. 6A). qPCR analysis showed that suppression of *CsABF2* led to a significant decrease in the transcript levels of *CsSGR1*, which is the key gene related to chlorophyll degradation, and *CsNYC1*, while the expression of *CsPAO* remained largely unchanged (Fig. 6B), suggesting that *CsSGR1* may be directly regulated by *CsABF2*. In the pathway of phenylpropanoid biosynthesis, the *CsPALa* gene also exhibited *CsABF2*-dependent expression (Fig. 6C). Moreover, the expression of genes involved in anthocyanin biosynthesis, including *CsMYB6c*, *CsF3′H*, *CsDFR*, and *CsANS*, was significantly regulated (Fig. 6D).

To elucidate the specific regulatory role of *CsABF2* in chlorophyll degradation, phenylpropanoid metabolism, and anthocyanin biosynthesis, the promoter regions of *CsSGR1*, *CsPALa*, *CsMYB6c*, *CsF3′H*, *CsDFR*, and *CsANS* were cloned and inserted into the pGreenII 0800 vector for dual-luciferase reporter assays. The results demonstrated that *CsABF2* significantly enhanced the promoter activities of *CsSGR1*, *CsPALa*, and *CsMYB6c*, whereas it had no significant effect on the transcriptional activation of *CsF3′H*, *CsDFR*, or *CsANS* (Fig. 6E–H). These findings suggest that *CsABF2* plays a key upstream role in regulating chlorophyll degradation and anthocyanin metabolism, thereby contributing to leaf coloration and metabolic reprogramming under low-temperature conditions. In the flavonoid biosynthetic pathway, R2R3-MYB, bHLH, and WD40 form a MYB-bHLH-WD40 (MBW) complex that serves as a core regulatory module [43, 44]. Co-expression assays involving *CsMYB6c*, the *bHLH* transcription factor *CsTT8-2*, and *WD40* showed that the promoter activities of *CsF3′H*, *CsDFR*, and *CsANS* were significantly upregulated by 11.59-fold, 8.43-fold, and 13.16-fold, respectively (Fig. 6I–N). These results indicate that *CsABF2* regulates the expression of downstream structural genes *CsF3'H*, *CsDFR*, and *CsANS* by activating *CsMYB6*, thereby promoting anthocyanin biosynthesis.

## Discussion

In early spring, low temperatures in high-altitude regions inhibit chlorophyll biosynthesis while promoting anthocyanin accumulation in tea plants. This results in newly sprouted tender leaves displaying tip yellowing or a uniform yellow or reddish hue across the entire leaf. These symptoms gradually fade as the leaves mature. Green tea made from such fresh leaves typically features a refreshing floral fragrance.

The yellowing observed on young, tender leaves of tea seedlings from each cultivar at the high-altitude BY and BM in Xizang (including the YG and LY tea gardens) differs from the spring leaf symptoms of tea plants grown at high altitudes in the mountainous areas of Anhui. In addition to yellowing at the leaf tips and margins, yellow spots appear on green leaves (Fig. 1E). The upper leaves exhibit uneven yellowing, while the lower leaves exhibit signs of browning. Observations indicate that these yellowing and browning symptoms gradually subside as temperatures rise (Fig. 1F). It is speculated that although the daytime maximum temperatures in these regions exceed the critical threshold for tea bud sprouting (10°C), the nighttime minimum temperatures remain in the low-temperature range above 0°C. This condition renders the newly sprouted tea leaves more vulnerable to cold stress.

To verify this hypothesis, tea plant samples were subjected to controlled temperature treatments: constant 4°C, constant 10°C, and a day/night temperature variation of 10°C / 4°C. Data analysis revealed that the day/night temperature fluctuation had a significant impact on the extent of cold injury and the progression of bud development, with lasting effects on the subsequent bud growth (Figs 1G–I). These findings further confirm that tea plants are particularly sensitive to cold stress during the sprouting stage [13, 45, 46]. Based on this, it is recommended that when introducing tea cultivars to high-latitude or high-altitude regions, priority should be given to those with cold tolerance or a later sprouting period.

This study utilized transcriptome sequencing to investigate the DEGs associated with the response of tea plants to cold stress. The analysis revealed that DEGs from both transcriptome datasets were predominantly enriched in pathways related to chlorophyll metabolism and the biosynthesis of phenylpropanoids and phenolic compounds (Figs 2 and 3). It is worth noting that a cold-induced *bZIP* transcription factor, *CsABF2*, was identified as a key candidate potentially involved in regulating leaf yellowing, lignin biosynthesis, and the production of phenolic compounds (Figs 4 and 5).

As a highly conserved protein family in eukaryotes, *bZIP* transcription factors are composed of a basic DNA-binding domain and a leucine zipper dimerization domain [47]. In the model plant *A. thaliana*, 75 *AtbZIP* transcription factors have been classified into 13 subfamilies, designated A to I, as well as S, M, K, and J. Among them, ABF transcription factors belong to subfamily A of the bZIP family [47]. Studies have shown that these transcription factors are crucial in the ABA signaling and are especially important in plant responses to abiotic stresses, including in tea plants [35, 36, 48]. Molecular experiments have demonstrated that *ABF2*, *ABF3*, and *ABF4* in *A. thaliana* can directly bind to and activate the promoters of *NYE1*, *PAO*, and *NYC1*, thereby participating in ABA-mediated chlorophyll degradation [34]. In AsODN interference experiments in tea plants, suppression of *CsABF2* expression led to a significant decrease in the expression of chlorophyll degradation-related genes *CsSGR1* and *CsNYC1* (Fig. 6A and B), suggesting that *CsSGR1* is likely transcriptionally regulated by *CsABF2*. Promoter activity assays further indicated that the *CsSGR1* promoter is positively regulated by *CsABF2* (Fig. 6F). These findings collectively confirm that *CsABF2* plays a regulatory role in chlorophyll degradation under low-temperature conditions.

**Figure 7:**
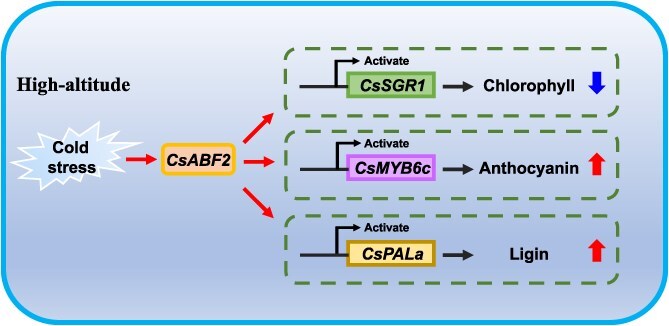
Schematic diagram of *CsABF2* regulation under low-temperature stress.

There is increasing evidence that *ABFs* are involved in regulating the biosynthesis of anthocyanins and other phenolic compounds. In *Litchi chinensis* Sonn., members of the bZIP family have been shown to play dual roles in pigment metabolism during fruit ripening, *LcABF1/2* promote chlorophyll degradation by activating chlorophyll catabolic genes such as *PAO* and *SGR*, while *LcABF3* influences anthocyanin accumulation by modulating the expression of the anthocyanin-related transcription factor *LcMYB1* [49]. In addition, the expression of *VvABF2* has been positively correlated with the biosynthesis of stilbenes, secondary metabolites that are crucial for plant defense [50]. AsODN experiments in tea plants further confirmed that *CsABF2* not only upregulates the expression of the key lignin biosynthetic gene *CsPALa*, but also activates the promoter activity of CsMYB6c, a transcription factor regulating anthocyanin biosynthesis (Fig. 6G and H). In summary, these findings demonstrate that *CsABF2*, which is induced by low temperature, not only facilitates chlorophyll degradation but also promotes lignin and anthocyanin biosynthesis in tea plants. This integrated regulatory mechanism is summarized in a proposed model (Fig. 7).

## Materials and methods

### Low-temperature treatment of tea seedlings

To assess how climatic conditions at different altitudes affect the growth of tea plants, twenty commercially available one-year-old tea cultivars were planted in the spring of 2022 at three locations in southeastern Xizang: Medog (MD, 1200 m), Zayü (ZY, 1720 m), and the high-altitude Layue in Bayi District (BY, 2600 m) and Yigong (YG,2200 m). After two years of cultivation, the growth and budburst of the seedlings were monitored in the following spring. Leaves exhibiting symptoms of cold damage were collected for transcriptome sequencing.

To simulate the impact of low temperatures in early spring on the growth of tea plants, three day/night temperature regimes were established in an artificial climate chamber: 4°C/4°C, 10°C/4°C, and 10°C/10°C. For each treatment, 90 one-year-old cold-resistant tea plants (*Camellia sinensis* cv. Zhongbai 1) were used. The seedlings were subjected to the low-temperature treatments for 15 days, followed by 30 days of cultivation under normal conditions at 25°C/20°C. Seedling growth and budburst were recorded after both the low-temperature treatment and the subsequent period at normal temperature.

To investigate the effects of low temperature on gene expression in tea plants, seedlings were exposed to two temperature regimes in the climate chamber: 25°C/20°C and 10°C/4°C (day/night). After 15 days of treatment, fresh leaf samples were collected for transcriptome sequencing and other analyses. *Nicotiana benthamiana* used in genetic transformation were grown in an artificial climate chamber under a 16-hour light/8-hour dark photoperiod at a constant temperature of 22°C.

### Meteorological data in southeastern Xizang

The data (1901–2023) for monthly mean temperature, monthly maximum temperature, and monthly minimum temperature, each at a 1-km spatial resolution, were obtained from the National Tibetan Plateau/Third Pole Environment Data Center (http://data.tpdc.ac.cn) and visualized using ArcMAP10.8.1. To monitor local temperature fluctuations, compact weather stations (model NHQXZ601, Wuhan Zhongke Nenghui Technology Development Co., Ltd.) were installed in tea gardens at MD, ZY, and BY, recording temperature changes from February to June 2024.

### Transcriptome and qRT-PCR analysis

Fresh tea leaf samples were submitted to Guangzhou Genedenovo Biotechnology Co., Ltd. for transcriptome sequencing, with three biological replicates per sample. Sequencing was conducted using the Illumina NovaSeq 6000 platform. The resulting paired-end reads were aligned to the reference genome of tea plants (http://tpia.teaplants.cn/) using HISAT2 with default settings. Gene expression was quantified using RSEM and expressed as fragments per kilobase of transcript per million mapped reads. DEGs were identified using the DESeq2 package, with genes meeting the criteria of FDR < 0.05 and |log₂FC| > 1 considered significantly differentially expressed.

NCBI database and Primer 5.0 were used to design qPCR primers for the target gene (Table S7). Total RNA was extracted from tea leaves using the MiniBEST Plant RNA Extraction Kit, following the manufacturer’s instructions. The concentration and quality of the extracted RNA were assessed using a microvolume spectrophotometer and agarose gel electrophoresis, respectively. Reverse transcription was conducted using the PrimeScript™ RT Reagent Kit to synthesize cDNA from the RNA samples. The resulting cDNA was diluted with RNase-free water to a final concentration of 250 ng/μl for use in qRT-PCR analysis. The *CsGAPDH* gene was used as an internal reference, and the relative expression of the target genes was calculated using the 2^−ΔΔCt^ method.

### Gene cloning and promoter cis-acting element prediction

Using cDNA from tea plant samples as the template, the target gene was amplified by PCR with a high-fidelity polymerase. The PCR product was then ligated into a cloning vector, and the inserted sequence was verified by DNA sequencing. The cloned target gene is used for subsequent experiments. To clone and analyze promoter sequences of the genes related to tea plants, chromosomal location for *CsABF2*, *CsF3′H*, *CsDFR*, *CsANS*, *CsSGR1*, *CsPALa*, and *CsMYB6c* was first retrieved from the published genomes of tea plants. Primers were designed based on the sequence within 2000 bp upstream of the start codon. Genomic DNA was extracted from tea leaves with a plant genomic DNA kit and used as the template for promoter cloning. The amplified promoter fragments were then verified by DNA sequencing. Primers were designed and synthesized based on the full-length coding sequence (Table S7).

The PlantCare website (http://bioinformatics.psb.ugent.be/webtools/plantcare/html/) was utilized to predict the cis-acting elements of promoters [51]. Subsequently, cis-acting elements associated with transcription factors were screened, and these cis-acting elements were visualized and analyzed using the TBtools software [52].

### Phylogenetic tree

A phylogenetic tree of the bZIP family in tea plants was constructed using the neighbor-joining (NJ) method in MEGA7 software [53], with bootstrap analysis performed using 1000 replicates. Gene names were assigned following the nomenclature convention of *A. thaliana*.

### Transient gene silencing in tea plants using antisense oligodeoxynucleotides

AsODN sequences targeting candidate genes were designed using Soligo [54] (https://sfold.wadsworth.org/cgi-bin/index.pl), while sense oligodeoxynucleotide (sODN) sequences were used as controls. The young shoots of tea plants were employed for AsODN treatment. A 100-μM solution of AsODN or sODN was injected into the abaxial side of the third leaf using a syringe. The treated seedlings were then incubated in an artificial climate chamber, and samples were collected after 48 hours for the analysis of gene expression. All primer sequences are listed in Table S7.

### Histochemical GUS staining

The promoters of the candidate genes were cloned into the pCAMBIA1304 vector to replace the CaMV35S promoter. The primers used are listed in Table S7. *Nicotiana benthamiana* leaves were incubated at 37°C for 12 hours, followed by decolorization in 75% ethanol. The ethanol was changed every 3 hours and replaced three times in total. After decolorization, the leaves were collected and photographed. For each treatment, at least three independent transgenic seedlings were analyzed, and representative results are shown.

### Dual-luciferase reporter assay

The promoter activation assay was conducted according to the method described by Jiao [44] with a minor modification. Promoter sequences of candidate genes were cloned into the pGreenII 0800 vector, and transcription factor sequences were cloned into the pGreenII 62-SK vector using a one-step rapid cloning kit. After sequence verification, the plasmids were introduced into the *Agrobacterium tumefaciens* strain GV3101. Equal volumes of *Agrobacterium* cultures carrying the promoter and transcription factor constructs were mixed and co-infiltrated into *N. benthamiana* leaves. Three days after infiltration, the leaves were sprayed evenly with D-luciferin sodium salt solution (50 μg/ml) and incubated in the dark for 5 min. Luminescence signals were then detected using a chemiluminescence imaging system. Based on the luminescence results, two leaf discs (10 mm in diameter) were punched from the infected areas and placed in 2-ml centrifuge tubes preloaded with zirconia beads. Samples were flash-frozen in liquid nitrogen and ground at 23 Hz for 60 s. After adding 100 μl of lysis buffer, samples were thawed on ice and lysed for 5 min. The lysates were centrifuged at 13 680 xg for 5 min at 4°C, and the supernatants were collected. Firefly and Renilla luciferase activities were measured according to the manufacturer’s protocol for the dual-luciferase assay kit.

### Chlorophyll content determination

The determination of chlorophyll content was conducted according to the previously reported method [55] with modifications. A total of 0.10 g of fresh tea leaves was finely chopped and homogenized, and then 10 ml of 95% ethanol extraction solution was added. The mixture was incubated in darkness at room temperature for 48 h. After extraction, the solution was centrifuged at 13 680 xg for 10 min and the supernatant was collected. The absorbance at 665, 649, and 470 nm was measured using a spectrophotometer, and the contents of chlorophyll a, chlorophyll b, and carotenoids were measured accordingly.

### Anthocyanin content determination

According to the previously published method [56, 57] with a minor modification. A total of 0.05 g of freeze-dried powder derived from fresh tea leaves was extracted with 1 ml of 80% methanol, which contained 0.5% hydrochloric acid, utilizing ultrasonic treatment. The extract was centrifuged at 13 680 xg for 10 min, after which the supernatant was collected. The residue underwent four additional extraction cycles, with all supernatants subsequently combined. The absorbance at 530 nm was measured using a spectrophotometer, and the anthocyanin content was calculated accordingly.

### Lignin content determination

Tea leaves were embedded and fixed using OCT compound (SAKURA, Japan), then sectioned transversely through the veins into 24-μm slices using a cryostat microtome (Leica CM3050S, Germany). Intact vein cross-sections were selected for staining, slide preparation, and microscopic observation. Each experiment was repeated three times. The transverse sections from both treatment and control groups were stained with phloroglucinol reagent and imaged using a stereomicroscope (ZEISS Axio Zoom.V16, Germany) to observe lignification in the vascular tissues.

The method for determining total lignin content was referenced from previous studies with slight modifications [58]. A total of 0.10 g of fresh tea leaf powder was weighed and extracted with 2 ml of 50% methanol in a water bath at 80°C for 5 h. The extract was centrifuged at 13 680 xg for 2 min, after which the supernatant was discarded and the pellet was collected and freeze-dried. A total of 0.004 g of the dried sample was transferred into a 10-ml stoppered test tube, followed by adding 2 ml of 25% acetyl bromide–glacial acetic acid solution (v/v) and 80 μL of 70% perchloric acid. The mixture was incubated in a 70°C water bath for 30 min. After incubation, 2 ml of 2 mol/L NaOH was added and mixed thoroughly, and the solution was brought to a final volume of 10 ml with glacial acetic acid. The absorbance of the extract was measured at 280 nm using a UV spectrophotometer, with glacial acetic acid as the blank control. Lignin content was quantified using a standard curve generated with commercially available alkali lignin.

### ABA extraction and content determination

The content of ABA was determined using a method adapted from existing literature [59]. Briefly, 100 mg of fresh leaf samples were ground into a powder in liquid nitrogen. Subsequently, 1 ml of ethyl acetate was added, and the mixture was ball-milled for 10 min, followed by centrifugation at 4°C for 10 min at 13 680 xg. The supernatant was then transferred to a 1.5-ml centrifuge tube, concentrated into a powder using a vacuum centrifuge, and completely dissolved in 50% methanol in water. The resulting solution was centrifuged again at 13 680 xg for 10 min and determination of ABA content in the supernatant using UPLC-QqQ-MS/MS (Agilent Technologies, Palo Alto, CA, USA). The mobile phases consisted of 0.1% (v/v) formic acid in water (solvent A) and 100% (v/v) methanol (solvent B). The elution gradient was set as follows: 0%–20% B for 6.5 min, followed by a balance period from 6.5 to 8.0 min, 95%–20% B from 8.0 to 8.1 min, and a final balance maintained from 8.1 to 10.0 min. The parent ion and their fragments of ABA was analyzed in negative mode by multiple reaction monitoring.

### Statistical analysis

Statistical analyses were conducted using one-way analysis of variance (ANOVA) in IBM SPSS Statistics for Windows, version 22.0 (IBMCorp., Armonk, N.Y., USA). Student’s *t*-test and one-way ANOVA was used to analyze the significance of differences between the two treatments. Graphs were generated using Origin (Pro), version 2024 (OriginLab Corporation, Northampton, MA, USA) and finalized using Adobe Illustrator 2021 for figure layout and export.

## Supplementary Material

Web_Material_uhaf279
