# Supplementary material for: Spring cold stress at high altitudes in southeastern Xizang activates CsABF2 to regulate chlorophyll degradation and phenolic biosynthesis in tea plants
Source: Hortic Res. 2025 Oct 22;13(1):uhaf279. doi: 10.1093/hr/uhaf279 (PMC12881854; doi:10.1093/hr/uhaf279)
Supplement: Web_Material_uhaf279 [file web_material_uhaf279.zip › Supplemental figures.docx]

**Spring cold stress at high altitudes in southeastern Xizang activates *CsABF2* to regulate chlorophyll degradation and phenolic biosynthesis in tea plants**

Yipeng Huang^1,^ *, Didi Jin^1,^ *, Tianming Jiao^1^, Zhenhong Wang^3^, Ting Jiang^1^, Lei Zhao^4^, Xiaolan Jiang^1^, Haiyan Wang^2^, Yajun Liu^2^, Yunsheng Wang^2, #^, Liping Gao^2, #^, Tao Xia^1, #^


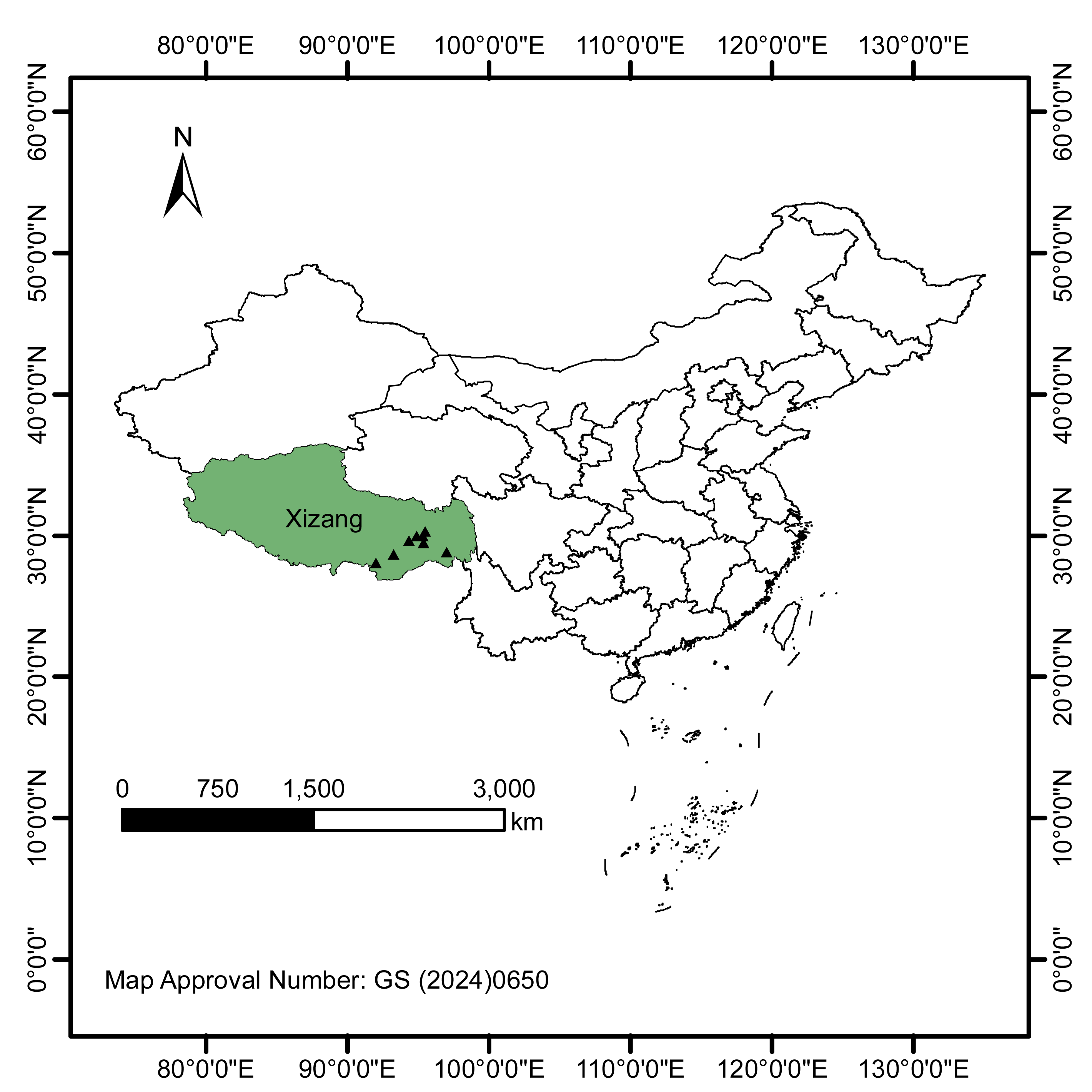


Fig. S1 Location of tea cultivation areas in Xizang within the national map of China

The map was obtained from the Tianditu website (<https://www.tianditu.gov.cn>; Map Approval Number: GS (2024)0650), and the black triangles indicate the main tea cultivation areas in Xizang.


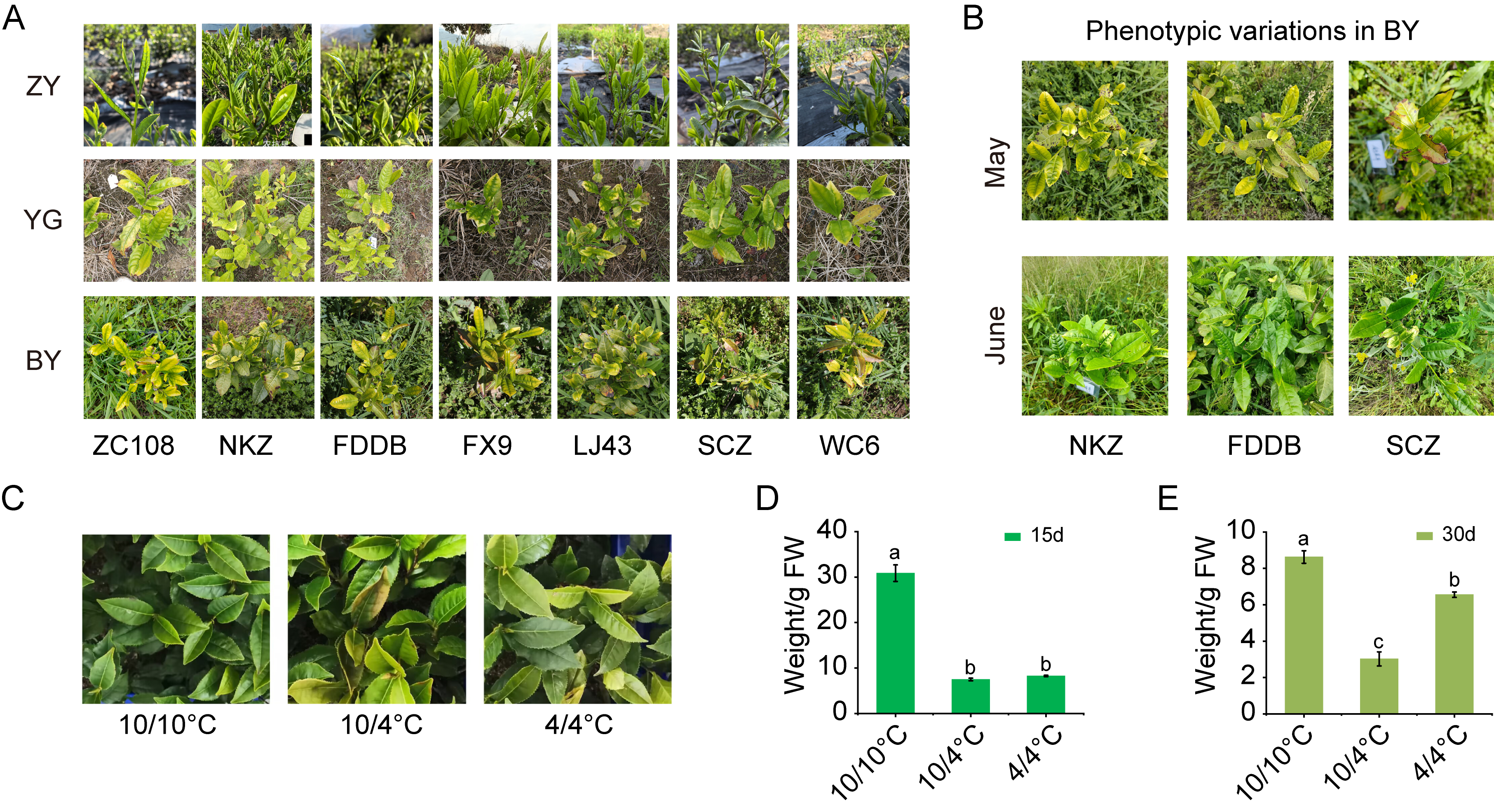


Fig. S2 Tea cultivation in southeastern Xizang and responses of tea seedlings to low-temperature treatment

(A) Phenotypes of tea seedlings from ZY, YG and BY in southeastern Xizang (photographed in mid-May). Tea cultivars grown in the region include C. sinensis cultivars Zhongcha 108 (ZC108), Nongkangzao (NKZ), Fuding Dabai (FDDB), Fuxuan No. 9 (FX9), Longjing 43 (LJ43), Suchazao (SCZ), and Wancha No. 6 (WC6); (B) Phenotypic variations of young tea leaves observed at BY tea garden in May and June; (C) Different low-temperature treatments applied to tea seedlings (light/dark temperatures), with light intensity of 10,000 lux and a 12 h light / 12 h dark photoperiod, administered for 30 days; (D) Fresh weight of young tea shoots (one bud with two leaves) after 15 days of recovery under optimal growth conditions (25/20°C, day/night) following low-temperature treatment; (E) Fresh weight of young tea shoots (one bud with two leaves) after 30 days of recovery optimal growth conditions (25/20°C, day/night) following low-temperature treatment. All data are presented as the mean ± standard deviation (SD) of three biological replicates (n = 3). Different letters indicate statistically significant differences at *P* < 0.05, based on Tukey’s honestly significant difference (HSD) test.
